# Association between ACE gene I/D polymorphism and knee osteoarthritis in a Chinese population

**DOI:** 10.1042/BSR20181713

**Published:** 2019-02-26

**Authors:** Genjun Chen, Shengping Hu, Zhen Lai, Binsong Qiu

**Affiliations:** 1 Department of Orthopedics, Zhejiang Chinese Medicine and Western Medicine Integrated Hospital/Hangzhou Red Cross Hospital, 208 East Huancheng Road, Hangzhou, Zhejiang, China; 2 Department of Orthopaedic Surgery, Zhejiang Provincial People’s Hospital, People’s Hospital of Hangzhou Medical College, 158 Shangtang Road, Hangzhou, Zhejiang, China

**Keywords:** angiotensin converting enzyme 2, case-control study, I/D polymorphism, knee osteoarthritis, meta-analysis

## Abstract

Osteoarthritis (OA) is a degenerative joint disease characterized by joint destruction with cartilage loss and occasional gross derangement of joint integrity. In recent years, several studies have reported the association between angiotensin-converting enzyme (ACE) gene insertion/deletion (I/D) polymorphism and knee OA. However, the results were conflicting. To determine the association between ACE gene I/D polymorphism and knee OA, we conducted a hospital-based case–control study with 282 knee OA cases and 316 controls to investigate the association between ACE gene I/D polymorphism and knee OA susceptibility in a Chinese Han population. The present study found that DD genotype or D allele carriers of ACE gene I/D polymorphism increased the risk of knee OA. Stratification analyses of sex, age, and body mass index (BMI) showed significant associations amongst the groups of females, ≥55 years, and abnormal BMI. In addition, the present study made analysis between ACE I/D polymorphism and some clinical features of OA, and found DD genotype of I/D polymorphism was associated with arthralgia. Furthermore, we undertook a meta-analysis together with the present study between this single nucleotide polymorphism (SNP) and knee OA risk. This meta-analysis found that ACE gene I/D polymorphism was associated with increased risk for OA. Stratification analysis of ethnicity in this meta-analysis indicated that I/D polymorphism increased the risk of knee OA amongst the Asians and Caucasians. In conclusion, this case–control study and meta-analysis suggest that ACE gene I/D polymorphism is associated with increased risk for knee OA.

## Introduction

Osteoarthritis (OA) is a degenerative joint disorder resulting in destruction of articular cartilage, osteophyte formation, and subchondral bone sclerosis [1]. OA is a polygenic disease and its pathogenesis is influenced by several environmental factors such as ageing, hormones, mechanical factors, and lifestyle. Genetic factors account for 50% of the risk of OA development [2] and prior research suggests that OA is primarily influenced by genetic risk factors due to common population polymorphisms in multiple genes [3–6]. Therefore, candidate gene studies may provide insight into OA development.

Angiotensin-converting enzyme (ACE) gene is localized on chromosome 17 and contains a polymorphism based on the presence (insertion, I) or absence (deletion, D) within intron 16, of a 287-bp Alu repeat sequence within intron 16, resulting in three different genotypes: DD and II homozygous and ID heterozygous. Plasma ACE levels vary with polymorphism. The role of ACE polymorphism has been investigated as a risk factor for several diseases such as inflammatory and immune-related disorders [7], particularly the rheumatic and autoimmune diseases.

Several studies have investigated the relationship between OA and the ACE I/D polymorphism [7–12], but the results were contradictory. As gene pools, lifestyle, and gene–environment interactions vary amongst the populations, the risk shall not be supposed as identical in every population with respect to genotypes. Therefore, we conducted this case–control study to investigate the association between ACE gene I/D polymorphism and knee OA susceptibility in a Chinese Han population. In addition, we performed a meta-analysis together with the present study to verify the relationship between this single nucleotide polymorphism (SNP) and knee OA risk comprehensively.

## Materials and methods

### Study population

A hospital-based case–control design was used in the present study. A total of 282 knee OA patients and 316 healthy controls were selected from Zhejiang Provincial People’s Hospital, People’s Hospital of Hangzhou Medical College between February 2014 and May 2017. The diagnosis of knee OA fulfilled the American College of Rheumatology criteria (1987) [13]. Inclusion criteria were: (i) any symptom and/or sign of knee OA, (ii) no evidence for any other form of arthritis, and (iii) informed consent obtained. Participants who had undergone knee surgery, and those with any systemic inflammatory or autoimmune disorder, or any type of malignant or chronic illness were excluded from the present study. Control subjects were consecutively selected amongst people without personal and family history of knee OA and were matched for age (±5 years) and sex. The demographic, lifestyle, and clinical characteristics of all patients and controls, such as age, gender, body mass index (BMI: kg/m^2^), and K-L grade were collected from medical records. This case–control study was approved by the Ethics Committee of Zhejiang Provincial People’s Hospital, People’s Hospital of Hangzhou Medical College and performed according to *Declaration of Helsinki*. All patients provided written informed consent prior to participation.

### Genomic DNA extraction and genotyping

Peripheral blood samples were taken from all patients and controls, and stored in EDTA tubes. DNA was extracted from blood examples using the QIAamp DNA Blood Mini Kit (Qiagen, Hilden, Germany). The genotypes of ACE gene polymorphism were determined by PCR using primers and conditions described previously [8,12]. The PCR program included the following steps: initial denaturation at 95°C for 5 min; 35 cycles of denaturation at 95°C for 30s, annealing at 58°C for 30 s, and extension at 72°C for 30s and a final extension at 72°C for 10 min. To control the quality of genotyping, two independent investigators interpreted images of each gel, and at least 10% of samples were randomly selected for repeated genotyping.

### Statistical analysis

Hardy–Weinberg equilibrium (HWE) of the SNP genotypes was analyzed by the goodness-of-fit chi-square (χ^2^) test to compare the observed and expected genotype frequencies amongst controls. Association between ACE gene I/D polymorphism and knee OA risk was assessed by logistic regression with odds ratio (OR) and 95% confidence interval (CI). χ^2^ test was used to assess the difference in the demographic characteristics, variables, and the genotypes of the ACE gene I/D polymorphism. Above statistical analyses were performed on SAS software package (ver. 9.1.3; SAS Institute, Cary, NC, U.S.A.) with the significant level at *P*<0.05. To fully investigate the association of ACE gene I/D polymorphism with knee OA, we also conducted a meta-analysis which was performed using the Stata 11.0 software (StataCorp, College Station, TX, U.S.A.).

## Results

### Clinical information of the study population

The characteristics of the subjects in the case and control groups are summarized in Table 1. Cases and controls were well matched in terms of age and gender (*P*=0.593 and *P*=0.328, respectively), and no significant differences in gender and age were observed between the OA patients and controls. A significant association was found in the subgroup of BMI. In addition, disease severity in the knee OA cases and controls was assessed via the Kellgren-Lawrence grade (K-L grade). The controls and cases were concentrated in grade 0–4. Other characteristics of the subjects are presented in Table 1.

**Table 1:** Characteristics of subjects with knee OA and control subjects

Variable	Patients (*n*=282)	Controls (*n*=316)	*P*-value
Age (years)	54.34 ± 4.55	54.62 ± 5.45	0.500
Number of male/female	75/207	77/239	0.532
BMI (kg/m^2^)	27.42 ± 3.34	25.00 ± 3.44	<0.001
Onset age	46.48 ± 6.52		
Duration of OA	7.56 ± 4.16		
ESR, mm/h	13.65 ± 4.83	14.20 ± 5.00	0.174
CRP, mg/l	2.50 ± 0.89	1.66 ± 0.46	<0.001
Symptoms of patients			
Arthralgia	219 (77.7%)		
Arthroncus	186 (66.0%)		
Muscle atrophy	31 (11.0%)		
Joint deformity	23 (8.2%)		
Kellgren–Lawrence grade		—	—
1	62 (22.0%)	—	—
2	141 (50.0%)	—	—
3	65 (23.0%)	—	—
4	14 (5.0%)	—	—

Abbreviations: CRP, C-reaction protein; ESR, erythrocyte sedimentation rate.

### Association between ACE gene I/D polymorphism and knee OA risk

The genotype distributions of ACE gene I/D polymorphism in all subjects are delineated in Table 2. Genotype distributions for rs2292832 polymorphism in the controls conformed to the HWE (*P*=0.802). Logistic regression analyses revealed that the DD genotype or D allele carriers of I/D polymorphism was significantly associated with increased risk of knee OA (DD compared with ID+II: adjusted OR = 1.83, 95% CI = 1.15–2.37, *P*=0.007; ID+DD compared with II: adjusted OR = 1.65, 95% CI = 1.15–2.37, *P*=0.007; D compared with I: OR = 1.47, 95% CI = 1.16–1.85, *P*=0.001; Table 2). We also conducted stratification analyses of sex, age, and BMI. We obtain significant associations amongst the groups of females, ≥55 years, abnormal BMI (Table 3). Furthermore, we investigated the association between ACE I/D polymorphism and clinical features of OA and found DD genotype of this SNP was associated with arthralgia (Table 4).

**Table 2:** Logistic regression analysis of associations between ACE I/D polymorphism and risk of knee OA

Genotype	Cases^1^ (*n*=282)		Controls^1^ (*n*=316)		OR (95% CI)	*P*	Adjusted OR (95% CI)^2^	Adjusted *P*^2^
	*n*	%	*n*	%				
II	39	14.0	67	21.3	1		1.0	
ID	128	45.9	157	49.8	1.40 (0.89, 2.22)	0.150	1.55 (0.95, 2.54)	0.077
DD	112	40.1	91	28.9	**2.11 (1.31, 3.42)**	0.002	** 2.28 (1.37, 3.82)**	0.002
DD compared with ID+II	112/167	40.1/59.9	91/224	28.9/71.1	**1.65 (1.17, 2.32)**	0.004	**1.83 (1.15, 2.90)**	0.011
ID+DD compared with II	240/39	86.0/14.0	248/67	79.7/21.3	**1.66 (1.08, 2.56)**	0.021	**1.65 (1.15, 2.37)**	0.007
Allele frequency								
I	206	36.9	291	46.2	1			
D	352	63.1	339	53.8	**1.47 (1.16, 1.85)**	0.001		

Bold values are statistically significant (*P*<0.05).

1 The genotyping was successful in 279 cases and 315 controls.

2 Adjusted for age, sex, and BMI.

**Table 3:** Stratified analyses between ACE I/D polymorphisms and the risk of OA

Variable	Case/control			ID compared with II	DD compared with II	DD compared with ID+II	DD+ID compared with II
	II	ID	DD	OR (95% CI); *P*	OR (95% CI); *P*	OR (95% CI); *P*	OR (95% CI); *P*
Sex							
Male	13/14	35/34	26/28	1.10 (0.46, 2.70); 0.821	1.00 (0.40, 2.52); 1.000	0.93 (0.48, 1.81); 0.828	1.06 (0.46, 2.44); 0.892
Female	26/53	93/123	86/63	1.54 (0.90, 2.65); 0.117	**2.78 (1.57, 4.92); <0.001**	**2.02 (1.35, 3.01); <0.001**	**1.96 (1.18, 3.27); 0.010**
Age (years)							
<55	18/32	65/76	61/52	1.52 (0.78, 2.96); 0.217	**2.09 (1.05, 4.14); 0.036**	1.53 (0.96, 2.43); 0.076	1.75 (0.93, 3.29); 0.081
≥55	21/35	63/81	51/39	1.30 (0.69, 2.44); 0.422	**2.18 (1.10, 4.31); 0.025**	**1.81 (1.09, 2.99); 0.021**	1.58 (0.87, 2.88); 0.133
BMI							
Normal	5/22	17/55	14/34	1.36 (0.45, 4.14); 0.588	1.81 (0.57, 5.74); 0.313	1.44 (0.66, 3.15); 0.360	1.53 (0.53, 4.40); 0.427
Abnormal	34/45	111/102	98/57	1.44 (0.86, 2.42); 0.169	**2.28 (1.31, 3.95); 0.004**	**1.74 (1.17, 2.60); 0.006**	**1.74 (1.06, 2.84); 0.027**

Bold values are statistically significant (*P*<0.05).

**Table 4:** The association between ACE I/D polymorphism and clinical features of OA

Variables	Genotype			Heterozygote	Homozygote	Dominant	Recessive
	DD	ID	II	OR (95% CI); *P*	OR (95% CI); *P*	OR (95% CI); *P*	OR (95% CI); *P*
Onset age							
≤52	89	101	31	1.0			
>52	23	27	8	1.04 (0.43, 2.51); 0.938	1.00 (0.41, 2.47); 0.998	1.02 (0.44, 2.36); 0.964	0.98 (0.54, 1.76); 0.932
Arthralgia							
-	23	30	15	1.0			
+	89	98	24	1.82 (0.78, 4.21); 0.161	**2.43 (1.02, 5.78); 0.042**	2.07 (0.94, 4.60); 0.068	1.52 (0.85, 2.72); 0.158
Arthroncus							
-	41	41	14	1.0			
+	71	87	25	1.19 (0.56, 2.52); 0.653	0.97 (0.45, 2.07); 0.937	1.08 (0.53, 2.19); 0.833	0.85 (0.52, 1.41); 0.527
Muscle atrophy							
-	99	111	38	1.0			
+	13	17	1	5.82 (0.75, 45.22); 0.059	5.00 (0.63, 39.47); 0.094	5.43 (0.72, 41.01); 0.067	1.09 (0.51, 2.32); 0.829
Joint deformity							
-	101	118	37	1.0			
+	11	10	2	1.57 (0.33, 7.48); 0.570	2.02 (0.43, 9.52); 0.368	1.77 (0.40, 7.88); 0.446	1.41 (0.60, 3.31); 0.433
K-L grade							
1–2	84	92	26	1.0			
3–4	28	36	13	0.78 (0.36, 1.69); 0.532	0.67 (0.30, 1.47); 0.314	0.82 (0.40,1.70); 0.593	1.25 (0.72, 2.14); 0.427

Bold values are statistically significant (*P*<0.05).

### Results of false-positive report probability analysis for significant findings

We preset 0.2 as the false-positive report probability (FPRP) threshold. As shown in Table 5, at the prior probability of 0.1, the significant findings for the ACE I/D polymorphism remained unstable in the allele, dominant, homozygote, and heterozygote models. Moreover, the same effects were also seen in the stratification analyses of population-based control (PB), HWE-positive, and Asian groups.

**Table 5:** FPRP values for associations between the ACE I/D polymorphism and OA risk

Variables	OR (95% CI)	*P*-value	Power	Prior probability				
				0.25	0.1	0.01	0.001	0.0001
D vs. I	**1.38 (1.03, 1.86)**	0.034	0.708	0.229	0.471	0.908	0.990	0.999
Caucasian	**2.12 (1.67, 2.68)**	**<0.001**	**0.622**	**<0.001**	**<0.001**	**0.002**	**0.015**	0.133
PB	** 1.52 (1.02, 2.27)**	0.041	0.474	0.355	0.622	0.948	0.995	0.999
Unclear OA	**2.12 (1.67, 2.68)**	**<0.001**	**0.622**	**<0.001**	**<0.001**	**0.002**	**0.015**	0.133
DD+ID compared with II	**1.70 (1.11, 2.60)**	0.014	0.500	0.285	0.545	0.929	0.993	0.999
Caucasian	**3.84 (2.17, 6.78)**	**<0.001**	**0.500**	**0.001**	**0.003**	**0.028**	0.222	0.741
PB	** 1.98 (1.10, 3.54)**	0.021	0.500	0.125	0.300	0.825	0.979	0.998
Unclear OA	**3.84 (2.17, 6.78)**	**<0.001**	**0.500**	**<0.001**	**<0.001**	**0.001**	**0.007**	0.067
DD compared with ID+II								
Caucasian	**1.84 (1.20, 2.84)**	0.006	0.500	0.037	0.103	0.559	0.927	0.992
Unclear OA	**1.91 (1.34, 2.72)**	<0.001	0.500	0.003	0.008	0.082	0.473	0.900
DD compared with II	** 1.84 (1.02, 3.30)**	<0.001	0.418	0.041	0.206	0.438	0.896	0.989
Caucasian	**4.32 (2.48, 7.54)**	**<0.001**	**0.500**	**<0.001**	**<0.001**	**<0.001**	**0.001**	0.005
Unclear OA	**4.32 (2.48, 7.54)**	**<0.001**	**0.500**	**<0.001**	**<0.001**	**<0.001**	**0.001**	0.005
HWE-negative	0.44 (0.21, 0.92)	0.029	0.500	0.127	0.304	0.828	0.980	0.998
Other methods	3.40 (1.36, 8.47)	0.009	0.500	0.052	0.140	0.642	0.948	0.995
ID compared with II	**1.59 (1.11, 2.27)**	0.011	0.500	0.079	0.204	0.739	0.966	0.997
Asian	**1.26 (1.02, 1.55)**	0.028	0.500	0.211	0.445	0.898	0.989	0.999
Caucasian	**3.26 (2.11, 5.06)**	**<0.001**	**0.500**	**<0.001**	**<0.001**	**<0.001**	**<0.001**	**0.003**
HWE-positive	**1.26 (1.02, 1.55)**	0.028	0.500	0.211	0.445	0.898	0.989	0.999
HWE-negative	**2.46 (1.10, 5.49)**	0.028	0.500	0.150	0.346	0.853	0.983	0.999
PB	**1.79 (1.09, 2.92)**	0.020	0.500	0.124	0.298	0.824	0.979	0.998
Knee OA	**1.24 (1.01, 1.52)**	0.038	0.500	0.234	0.479	0.910	0.990	0.999
Unclear OA	**3.26 (2.11, 5.06)**	**<0.001**	**0.500**	**<0.001**	**<0.001**	**<0.001**	**<0.001**	**0.003**

### General characteristics of included studies and quantitative analysis of this meta-analysis

Table 6 lists the characteristics of included studies exploring the associations between ACE gene I/D polymorphism and knee OA. Four Asian studies (including the present study), two Caucasian studies, and one Arabian study were identified in this meta-analysis. The Newcastle–Ottawa Scales (NOS) scores of all included studies ranged from 5 to 6 stars, suggesting that they were studies of high methodological quality.

**Table 6:** Characteristics of included studies

Author and year	Age		Sex (male/female)		SOC	Country	Ethnicity	OA TYPE	Case/ control	Genotyping method	HWE	NOS
	Case	Control	Case	Control								
Hong, 2003	58.6 ± 9.4	N/A	48/94	N/A	PB	Korea	Asian	Knee OA	142/135	PCR	0.117	6
Shehab, 2008	57.07 ± 9.15	N/A	13/102	N/A	HB	Kuwait	Arabian	Knee OA	115/111	PCR	<0.001	5
Bayram, 2010	54.16 ± 1.20	44.6 ± 2.00	38/102	17/43	PB	Turkey	Caucasian	Unclear OA	140/60	PCR	0.013	5
Inair, 2013	58.04 ± 10.87	53.03 ± 12.88	60/161	65/135	PB	Turkey	Caucasian	Unclear OA	221/200	PCR	0.003	6
Poornima, 2014	42.41 ± 8.11	42.17 ± 7.98	32/68	31/69	PB	India	Asian	Knee OA	100/100	PCR	0.480	5
Lin, 2016	74.9 ± 7.1	73.3 ± 6.6	194/253	206/217	PB	Taiwan	Asian	Knee OA	447/423	PCR	0.945	6
The present study	54.34 ± 4.55	54.62 ± 5.45	75/207	77/239	HB	China	Asian	Knee OA	282/316	MALDI-TOF-MS	0.963	6

Abbreviations: HB, hospital-based control; SOC, source of control.

**Figure 1:**
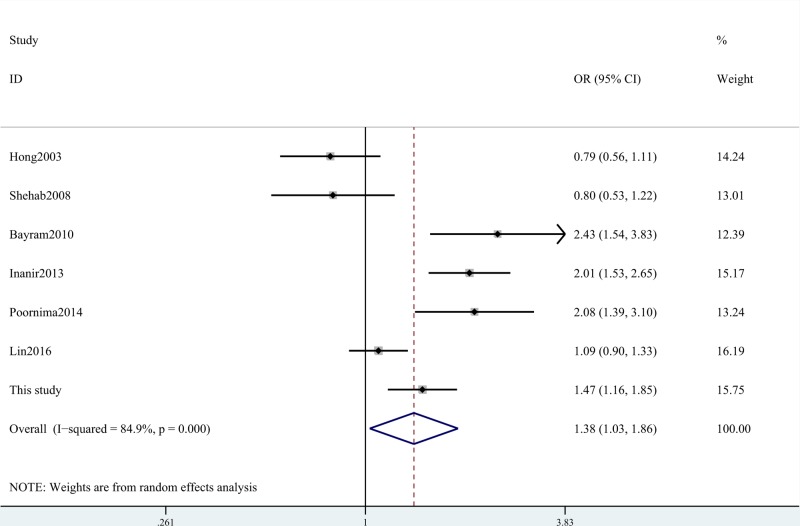
Forest plot shows OR for the associations between ACE gene I/D polymorphism and OA risk (D compared with I)

**Figure 2:**
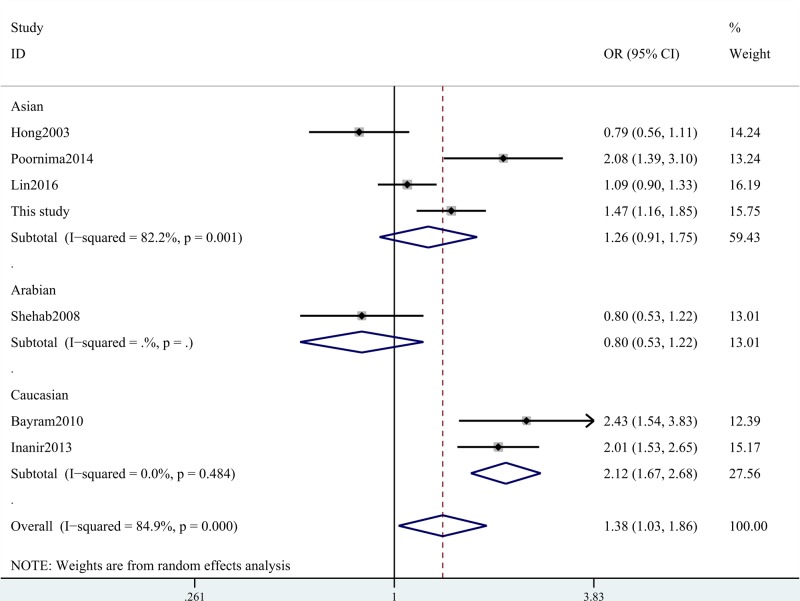
Stratification analysis by ethnicity shows OR for the association between ACE gene I/D polymorphism and OA risk (D compared with I)

**Figure 3:**
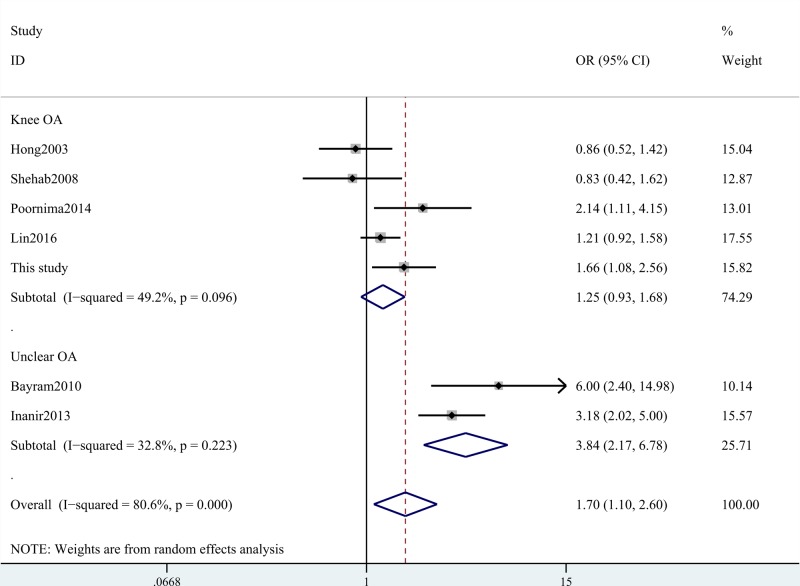
Stratification analysis by type of OA shows OR for the association between ACE gene I/D polymorphism and OA risk (DD+ID compared with II)

Our meta-analysis indicated that ACE gene I/D polymorphism was associated with the increased risk for knee OA (D compared with I: OR = 1.39, 95% CI = 1.03–1.86, *P*=0.031; DD+ID compared with II: OR = 1.70, 95% CI = 1.11–2.60, *P*=0.016; DD compared with II: OR = 1.84, 95% CI = 1.02–3.30, *P*=0.041; ID compared with II: OR = 1.59, 95% CI = 1.11–2.27, *P*=0.010; Table 7 and Figure 1). In the subgroup analysis of ethnicity, we found that this SNP increased OA risk amongst Asian and Caucasian populations (Table 7 and Figure 2). Stratification analysis by types of OA indicated that ACE gene I/D polymorphism increased the risk of knee OA and unclear OA (Table 7 and Figure 3). We also observed positive results in the Chinese groups. We did not obtain any different conclusions after eliminating the studies which did not meet the HWE, indicating that the data of this meta-analysis were trustworthy and stable.

**Table 7:** Meta-analysis of association between ACE I/D polymorphism and the risk of knee OA

Comparison	Category	Category	Studies	OR (95% CI)	*P*-value	*P* for heterogeneity
D compared with I	Total		7	**1.38 (1.03, 1.86)**	0.031	<0.001
	Ethnicity	Asian	4	1.26 (0.91, 1.75)	0.171	<0.001
		Arabian	1	0.80 (0.53, 1.22)	0.302	N/A
		Caucasian	2	**2.12 (1.67, 2.68)**	<0.001	0.484
	Population	Non-Chinese	5	1.45 (0.90, 2.33)	0.128	<0.001
		Chinese	2	1.26 (0.94, 1.68)	0.122	0.057
	HWE	HWE-positive	4	1.26 (0.91, 1.75)	0.171	0.001
		HWE-negative	3	1.58 (0.85, 2.95)	0.148	<0.001
	SOC	PB	5	**1.52 (1.02, 2.27)**	0.042	0.180
		HB	2	1.11 (0.62, 2.00)	0.719	0.152
	Types of OA	Knee OA	5	1.16 (0.86, 1.57)	0.321	0.091
		Unclear OA	2	**2.12 (1.67, 2.68)**	<0.001	<0.001
DD+ID compared with II	Total		7	**1.70 (1.11, 2.60)**	0.016	<0.001
	Ethnicity	Asian	4	1.33 (0.97, 1.83)	0.078	0.097
		Arabian	1	0.83 (0.42, 1.62)	0.578	N/A
		Caucasian	2	**3.84 (2.17, 6.78)**	<0.001	0.223
	Population	Non-Chinese	5	1.90 (0.94, 3.82)	0.073	<0.001
		Chinese	2	1.32 (1.05, 1.66)	0.016	0.220
	HWE	HWE-positive	4	1.33 (0.97, 1.83)	0.078	0.097
		HWE-negative	3	2.45 (0.86, 6.93)	0.093	0.001
	SOC	PB	5	**1.98 (1.10, 3.54)**	0.022	0.359
		HB	2	1.23 (0.63, 2.43)	0.548	0.161
	Types of OA	Knee OA	5	1.25 (0.93, 1.68)	0.141	0.054
		Unclear OA	2	**3.84 (2.17,6.78)**	<0.001	0.066
DD compared with ID+II	Total		7	1.34 (0.92, 1.94)	0.130	<0.001
	Ethnicity	Asian	4	1.27 (0.72, 2.22)	0.408	0.001
		Arabian	1	0.78 (0.45, 1.34)	0.365	N/A
		Caucasian	2	**1.84 (1.20, 2.84)**	<0.001	0.771
	Population	Non-Chinese	5	1.37 (0.79, 2.4)	0.264	<0.001
		Chinese	2	1.26 (0.73, 2.19)	0.407	0.041
	HWE	HWE-positive	4	1.27 (0.72, 2.22)	0.408	0.001
		HWE-negative	3	1.43 (0.80, 2.58)	0.228	0.024
	SOC	PB	5	1.42 (0.85, 2.36)	0.184	0.267
		HB	2	1.17 (0.56, 2.44)	0.677	0.230
	Types of OA	Knee OA	5	1.15 (0.71, 1.86)	0.562	0.233
		Unclear OA	2	**1.91 (1.34, 2.72)**	<0.001	<0.001
DD compared with II	Total		7	**1.84 (1.02, 3.30)**	0.041	<0.001
	Ethnicity	Asian	4	1.45 (0.75, 2.82)	0.268	0.001
		Arabian	1	0.78 (0.39, 1.56)	0.484	N/A
		Caucasian	2	**4.32 (2.48, 7.54)**	<0.001	0.265
	Population	Non-Chinese	5	2.05 (0.84, 5.02)	0.117	<0.001
		Chinese	2	1.49 (0.75, 2.93)	0.253	0.039
	HWE	HWE-positive	4	1.45 (0.75, 2.82)	0.268	0.001
		HWE-negative	3	2.60 (0.79, 8.55)	0.115	<0.001
	SOC	PB	5	2.14 (0.95, 4.83)	0.066	0.737
		HB	2	1.33 (0.50, 3.51)	0.569	0.403
	Types of OA	Knee OA	5	1.29 (0.73, 2.28)	0.377	0.322
		Unclear OA	2	**4.32 (2.48, 7.54)**	<0.001	0.038
ID compared with II	Total		7	**1.59 (1.11, 2.27)**	0.010	0.007
	Ethnicity	Asian	4	**1.26 (1.02, 1.55)**	0.034	0.794
		Arabian	1	1.01 (0.42, 2.41)	0.983	N/A
		Caucasian	2	**3.26 (2.11, 5.06)**	<0.001	0.326
	Population	Non-Chinese	5	**1.81 (1.01, 3.23)**	0.046	0.006
		Chinese	2	**1.29 (1.01, 1.64)**	0.040	0.673
	HWE	HWE-positive	4	**1.26 (1.02, 1.55)**	0.034	0.794
		HWE-negative	3	**2.46 (1.10, 5.49)**	0.028	0.038
	SOC	PB	5	**1.79 (1.09, 2.92)**	0.021	0.229
		HB	2	1.30 (1.12, 2.27)	0.199	<0.001
	Types of OA	Knee OA	5	**1.24 (1.01, 1.52)**	0.039	<0.001
		Unclear OA	2	**3.26 (2.11, 5.06)**	<0.001	<0.001

Abbreviations: HB, hospital-based control; SOC, source of control.

## Discussion

The present case–control study showed that the DD genotype or D carriers of I/D polymorphism was associated with significantly increased risk of knee OA in a Chinese Han population. Additionally, the present study obtained significant associations amongst the groups of females, ≥55 years, and abnormal BMI. Furthermore, the study found DD genotype of I/D polymorphism was associated with arthralgia. Using meta-analysis, we also found that ACE gene I/D polymorphism was associated with increased risk of OA. Similar results were replicated in the stratification analyses of ethnicity, HWE, SOC, and types of OA. In conclusion, this case–control study and meta-analysis found that ACE gene I/D polymorphism is associated with increased risk for knee OA.

In previous studies, ACE gene was found associated with rheumatic and autoimmune diseases. OA is an inflammatory disorder, which is associated with these proinflammatory cytokines [14]. Angiotensin II played a vital role in OA progression by modulating the synthesis of proinflammatory cytokines including tumor necrosis factor-alpha (TNF-α), CCL2 (C-C motif chemokine ligand 2), and IL-6 (Interleukin-6) [15]. Notably, levels of ACE in synovial fluid were significantly higher than control groups [16] in OA patients. Studies have demonstrated that the presence of D allele of I/D polymorphism is associated with higher levels of plasma ACE [17].

Several studies investigated the association between ACE gene I/D polymorphism and OA risk. Hong et al. [7] found ACE I/D polymorphism was a risk factor for early-onset knee OA in a Korean study. They indicated that I allele was associated with the radiographically severe and functionally poor OA [7]. In a subsequent study from Kuwait, Shehab et al. [12] revealed that there was no significant difference in the distribution of genotype or allele frequency between control subjects and OA groups. In addition, they did not find an association between any genotypes of I/D and clinical severity of OA [12]. Shehab et al. [12] showed a very high incidence of D-allele in Kuwaiti Arabs, while the Korean study [7] indicated the frequency of I-allele was significantly higher in early onset OA. The distinct distribution of allele frequency may explain the different findings of abovementioned two studies [7,12]. Poornima et al. [11] observed that I/D polymorphism was associated with increased risk of OA in an Indian population. Similar results were also obtained in two Turkish studies [8,9]. They [8,9] both suggested that DD genotype of the ACE gene I/D polymorphism increased the risk of knee OA in Turkish populations. Due to the conflicting findings, Lin et al. [10] conducted a case–control study and meta-analysis to validate the association of I/D polymorphism with OA risk. They did not find any significant association between I/D polymorphism and knee OA in the case–control study and meta-analysis [10]. It was noteworthy that they regarded the Turkish populations as Arabians. We thought that the Turkish populations should be divided into Caucasians. Moreover, the meta-analysis by Lin et al. [10] actually found a significant difference in the dominant model [OR: 1.72 (95% CI: 1.02 ± 2.90)], indicating that DD/ID genotype carriers had an increased risk for OA [10]. However, Lin et al. [10] regarded it as negative finding. Two previous meta-analyses investigated the association between this SNP and OA risk. Ai et al. [18] only found I/D polymorphism was associated with increased risk for Caucasians, but not in overall populations and Asians. In another meta-analysis, Shang et al. [19] suggested that ID genotype was weakly associated with OA risk. This meta-analysis (total 1447 cases and 1345 controls) indicated that ACE gene I/D polymorphism was associated with the increased risk of OA.

In the subgroup analysis of ethnicity, we found that ACE gene I/D polymorphism increased OA risk amongst Asian and Caucasian populations. However, no significant associations were obtained in the meta-analysis by Lin et al. [10]. In the meta-analysis of Ai et al. [18], they showed significant association only amongst Caucasians, which was different from that of this meta-analysis. This meta-analysis found that SNP was associated with increased risk for overall populations, including Caucasians and Asians. We also conducted the stratification analysis of types of OA, indicating that ACE gene I/D polymorphism increased the knee OA and unclear OA. In addition, we did not obtain any different conclusions after eliminating the studies which did not meet the HWE. Sensitivity analysis indicated that our results were more robust and stable. Furthermore, we performed stratification analyses of sex, age, and BMI, and found significant associations amongst the groups of females, ≥55 years, abnormal BMI, indicating that these exposure factors may play an important role in the interaction between this SNP and OA. We further made analysis between ACE I/D polymorphism and some clinical features of OA and found DD genotype of this SNP was associated with arthralgia.

Several potential limitations of this case–control study and meta-analysis should be considered. First, we cannot conduct the subgroups of some confounding factors due to the lack of corresponding data. Second, our results were based on unadjusted estimates for confounding factors. Third, the sample size of the present study was not large enough, which might make our work underpowered. Fourth, the sample sizes of Caucasians and Arabians were not large enough in this meta-analysis. Fifth, included studies were only involved in Asians, Caucasians, and Arabians, and studies amongst other racial groups are urgently needed. Sixth, the present study only focussed on one gene. Seventh, the present study with limited sample sizes could not provide sufficient evidence to draw a certain conclusion. Finally, we cannot conclude that I/D polymorphisms are susceptibility loci for other types of OA, highlighting the necessity for the further investigation of more types of OA.

In conclusion, this case–control study and meta-analysis found that ACE gene I/D polymorphism is associated with increased risk for knee OA. Further multi-center, well-designed studies with larger sample sizes that include gene–environment interactions assessment are warranted to confirm our findings.

## Abbreviations

ACE: angiotensin-converting enzyme
BMI: body mass index
CCL2: C-C motif chemokine ligand 2
CI: confidence interval
D: deletion
HWE: Hardy–Weinberg equilibrium
I: insertion
IL-6: Interleukin-6
K-L grade: Kellgren-Lawrence grade
OA: osteoarthritis
OR: odds ratio
PB: population-based control
SNP: single nucleotide polymorphism
TNF-α: tumor necrosis factor-alpha
